# Development of a Conjunctivitis Outpatient Rate Prediction Model Incorporating Ambient Ozone and Meteorological Factors in South Korea

**DOI:** 10.3389/fphar.2018.01135

**Published:** 2018-10-09

**Authors:** Jeong-Won Seo, Jong-Sang Youn, SeJoon Park, Choun-Ki Joo

**Affiliations:** ^1^Department of Ophthalmology, Hallym University, Dongtan Sacred Heart Hospital, Hwaseong-si, South Korea; ^2^Department of Environmental Engineering, Inha University, Incheon, South Korea; ^3^Department of Industrial and Management Engineering, Myongji University, Yongin-si, South Korea; ^4^Department of Ophthalmology and Visual Science, Catholic University of Korea, Seoul St. Mary's Hospital, Seoul, South Korea

**Keywords:** multi-level, conjunctivitis, ozone, prediction model, meteorology

## Abstract

Ozone (O_3_) is a commonly known air pollutant that causes adverse health effects. This study developed a multi-level prediction model for conjunctivitis in outpatients due to exposure to O_3_ by using 3 years of ambient O_3_ data, meteorological data, and hospital data in Seoul, South Korea. We confirmed that the rate of conjunctivitis in outpatients (conjunctivitis outpatient rate) was highly correlated with O_3_ (*R*^2^ = 0.49), temperature (*R*^2^ = 0.72), and relative humidity (*R*^2^ = 0.29). A multi-level regression model for the conjunctivitis outpatient rate was well-developed, on the basis of sex and age, by adding statistical factors. This model will contribute to the prediction of conjunctivitis outpatient rate for each sex and age, using O_3_ and meteorological data.

## Introduction

Air pollution is a significant global issue that has substantial effects on air quality, human health, earth hydrological cycle, and climate change (Correia et al., 2013; Lelieveld et al., 2015; Sicard et al., 2016; Duan et al., 2017). The Clean Air Act recommends that the U.S. Environmental Protection Agency (EPA) build National Ambient Air Quality Standards for “six criteria air pollutants,” which include particulate matter (PM), carbon monoxide (CO), sulfur dioxide (SO_2_), nitrogen dioxide (NO_2_), lead, and ozone (O_3_) (U. S. Environmental Protection Agency, 2010). The six criteria air pollutants are known to cause a wide range of health effects, including respiratory (Guan et al., 2016), cardiovascular (Franklin et al., 2015), eye (Szyszkowicz et al., 2018), and skin diseases (Eastham et al., 2018). Among the six criteria air pollutants, O_3_ is commonly known as the most toxic component produced by photochemical reactions in the atmosphere (Seinfeld and Pandis, 2006). Bell et al. (2004) revealed the relationship between O_3_ and short-term mortality in 95 communities in the United States.

Previous epidemiological studies have associated significant adverse human health effects by exposure to O_3_ (Fann et al., 2012). While much attention is focused on the effect of O_3_ on respiratory diseases (Sousa et al., 2013; Karakatsani et al., 2017; Stergiopoulou et al., 2018), less effort has been attached to discerning its role in eye disease. The effects of O_3_ on eye disease have been investigated in epidemiological studies (Hong et al., 2016; Hwang et al., 2016). Hong et al. (2016) studied the relationships of air pollutants (SO_2_, NO_2_, O_3_, PM_10_, PM_2.5_) and meteorological data with allergic conjunctivitis outpatients by using a retrospective registry study. However, that study had limitations in its analysis of the multi-level effect of air pollutants and meteorological data on conjunctivitis outpatient rate because it used the relationships between outpatients and individual factors. Hwang et al. found that dry eye disease outpatient rate was associated with high ozone concentration and low relative humidity, by using multivariable regression analysis.

The goal of this study was to develop a multi-level prediction model for conjunctivitis outpatient rate according to O_3_ and meteorological factors in Seoul, South Korea. Three years of O_3_ data, meteorological factors, and conjunctivitis outpatient rates in Seoul are reported. The subsequent discussion focuses on development and validation of a conjunctivitis outpatient prediction model with those data.

## Materials and methods

### Hospitalization data

Conjunctivitis outpatient statistic data between January 1, 2011 and December 31, 2013 in Seoul were obtained from the Korea Health Insurance Review and Assessment Service (KHIRAS) for research purpose. The KHIRAS provided number of ophthalmology outpatient based on diagnostic codes excluding patient personal information. In total, 97.2% of Korean residents receive Korea National Health Insurance Service (KNHIS) health insurance (Korean National Health Insurance Services, 2016). All hospitals in Korea are required to submit claim documents for medical services. We obtained data for 48,344 conjunctivitis patients, except waterborne and chronic conjunctivitis patients, based on disease code. The conjunctivitis outpatient rates of each age range and gender were calculated as the number of outpatients divided by the population, in order to normalize the data.

### Air pollutants and meteorological data

**Figure 1:**
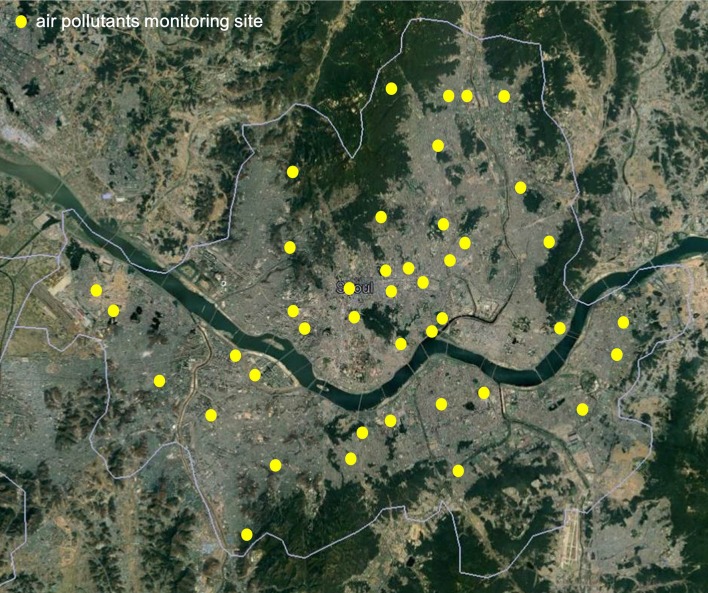
Air pollutants sampling sites in Seoul.

Hourly measurements of O_3_ were obtained for the years between January 1, 2011 and December 31, 2013 from 40 ground-based air pollutant monitoring sites operated by the city of Seoul, South Korea (Figure 1). To determine how meteorological factors are related to conjunctivitis outpatient rate, hourly temperature and relative humidity data were obtained at the collocated sites. We used weekly average data of patient visits and meteorological factors to avoid statistical errors due to no patient visits on weekends.

### Model development

**Figure 2:**
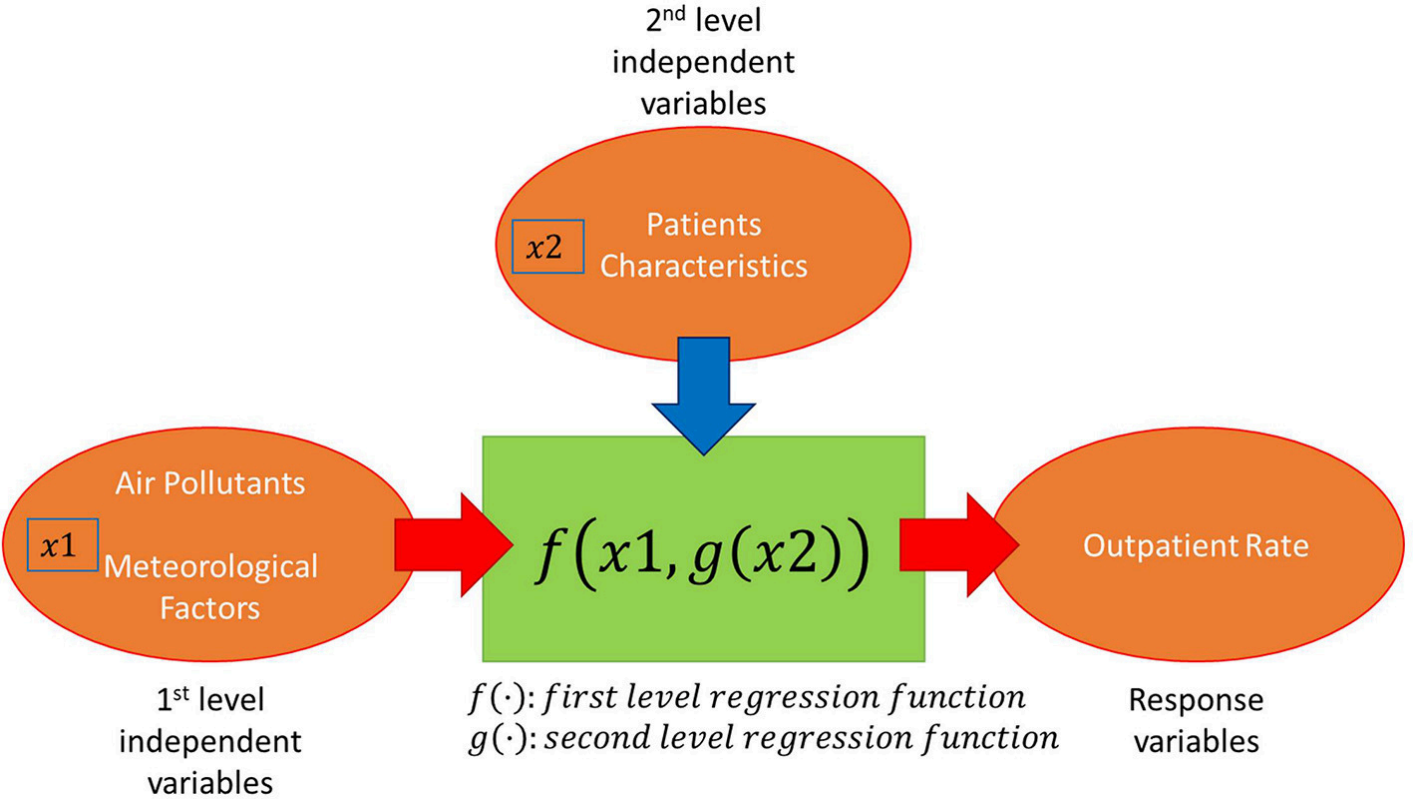
Structure of multi-level regression model for conjunctivitis outpatient rate.

A multi-level regression model (two-level regression model) was developed for the prediction of conjunctivitis outpatient rate. The structure of the model is shown in Figure 2. The level 1 regression model describes the relationship between level 1 independent variables and the conjunctivitis outpatient rate. Four air pollutants (PM_10_, NO_2_, SO_2_, and O_3_) and two meteorological factors (temperature and humidity) were considered as candidate level 1 model independent variables. Correlations between these factors and the conjunctivitis outpatient rate were calculated. PM_10_, NO_2_, and SO_2_ were removed from the level 1 regression model due to their negative correlations. The level 1 regression model was developed for each age range and gender. The shapes of the level 1 regression model were changed based on age range and gender. The coefficients of level 1 regression model can be explained by level 2 independent variables. An ANOVA was tested for the level 1 regression models and multi-level regression models. The detailed analysis and results are shown in the next section.

## Results and discussion

**Figure 3:**
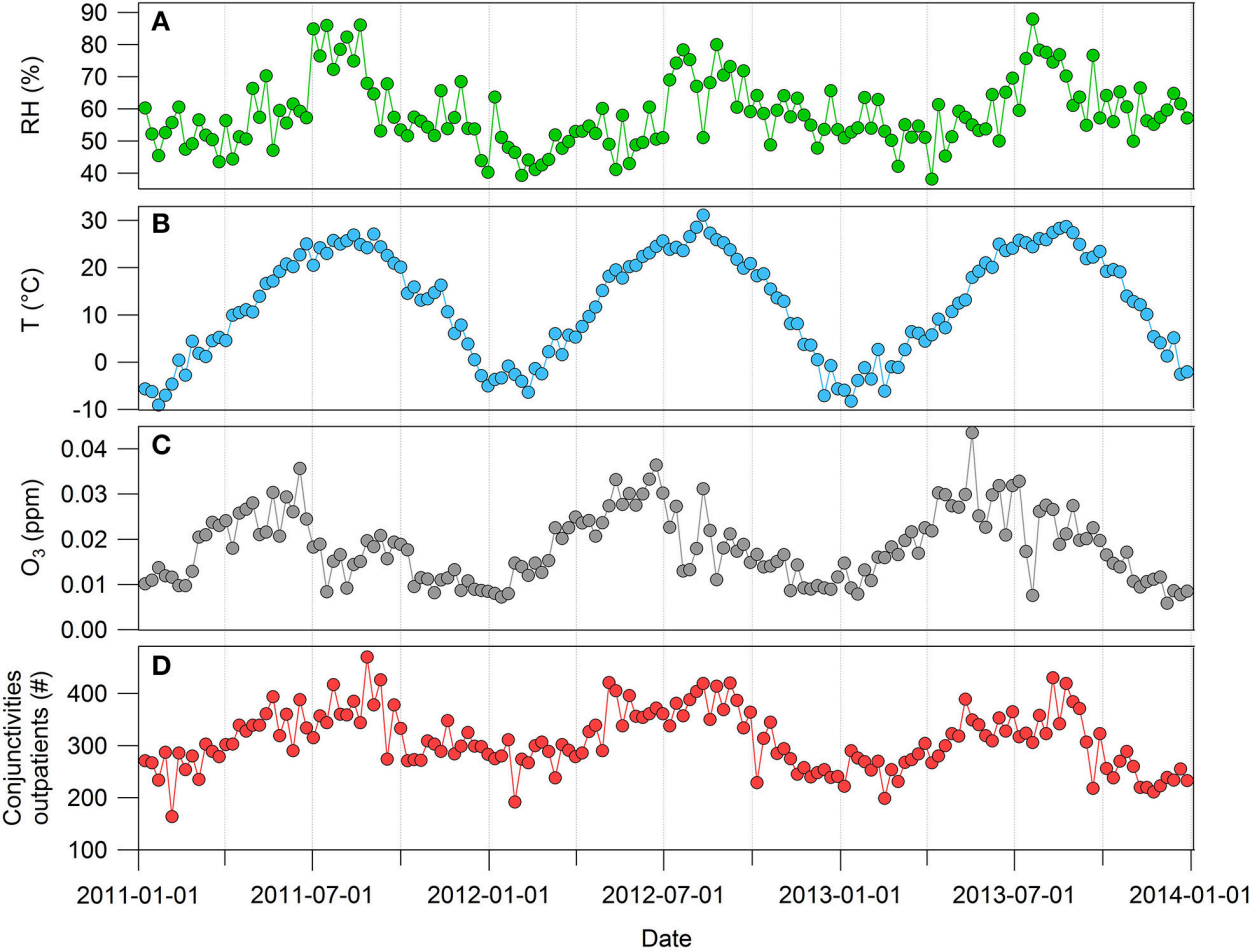
Weekly trends of **(A)** relative humidity (RH), **(B)** temperature (T), **(C)** O_3_, and **(D)** number of conjunctivitis outpatients.

Figure 3 shows the weekly trends of meteorological factors, O_3_, and conjunctivitis outpatient rates between 2011 and 2013. The highest and lowest seasonal averages of O_3_ concentrations from the sampling sites were 0.27 (April–June) and 0.12 ppm (October–December), respectively. The July–September data contained the highest values for temperature (24.7°C), humidity (70.7%), and number of conjunctivitis outpatients (359.5), while between January and March data had lowest values for temperature (−0.8°C), humidity (51.2%), and number of conjunctivitis outpatients (267.0). The number of conjunctivitis outpatients was positively correlated with the temperature (*R*^2^ = 0.72) and humidity (*R*^2^ = 0.29). The correlation coefficient between the number of conjunctivitis outpatients and O_3_ is 0.49. We developed a regression model based on the relationships between the number of conjunctivitis outpatients and other factors.

In previous research (Hong et al., 2016), the effect of each factor on conjunctivitis was examined individually. In contrast, in this study, regression models were developed with five independent factors, including temperature, humidity, O_3_, sex, and age, in order to consider these factors concurrently. First, the regression models for temperature, humidity, and O_3_ were developed, then sex and age factors were added by multi-level regression modeling. All regression models were developed by R 3.2.3 with the MASS library. The response variable and independent variables for the developed regression models were as follows:

*y*: outpatient rate per week (the number of outpatients per week/the population),
*X*_1_: average temperature per week + 20 (°C),
*X*_2_: average humidity per week (%),
*X*_3_: average O_3_ per week(ppm).

*y* is the response variable of the developed regression models; *X*_1_, *X*_2_, and *X*_3_ are the independent variables. In order to prevent negative values, the average temperature per week + 20 was used for *X*_1_, instead of the average temperature. Three simple regression models were developed, including the linear, linear + log, and linear + exponential models, with these response variable and independent variables (Kutner et al., 2004). The models are shown below:

**Model 1:** y = β_0_ + β_11_*X*_1_ + β_21_*X*_2_ + β_31_*X*_3_ + ε,
**Model 2:** y = β_0_ + β_11_*X*_1_ + β_12_ln(*X*_1_) + β_21_*X*_2_ + β_22_ln(*X*_2_) + β_31_*X*_3_ + β_32_ln(*X*_3_) + ε,
**Model 3:** y = β_0_ + β_11_*X*_1_ + β_12_exp(*X*_1_) + β_21_*X*_2_ + β_22_exp(*X*_2_) + β_31_*X*_3_ + β_32_exp(*X*_3_) + ε.

**Figure 4:**
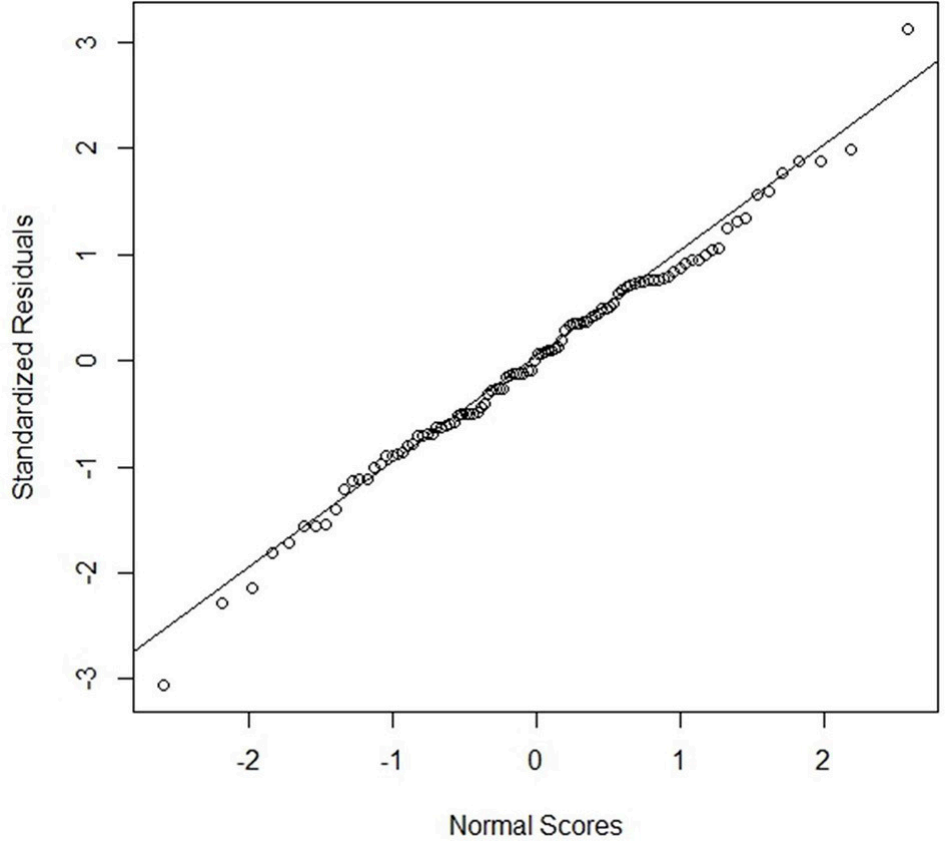
Normal probability plot for model 2.

The estimated coefficients of each model and the test results are shown in Table 1. One week for every 3 weeks over 156 weeks was randomly selected for only model validation (out-of-sample test). The other 2 weeks for every 3 weeks were used for model development and validation (in-sample test). All three models were significant based on their small p values. However, model 2 was the best model due to better *R*^2^ and Adjusted *R*^2^ for in-sample and out-of-sample tests. Figure 4 shows the normal probability plot for model 2. Most residuals in the graph are located near the diagonal line, which shows normality of residuals.

**Table 1:** Three regression models and their test results.

**Coefficients**	**Model 1**	**Model 2**	**Model 3**
β_0_	2.1E-05	8.1E-05	−4.2E-03
β_11_	3.6E-07	7.6E-07	3.6E-07
β_12_	–	−1.1E-05	1.7E-28
β_21_	−7.8E-08	1.6E-07	−7.0E-08
β_22_	–	−1.5E-05	−4.7E-45
β_31_	1.2E-04	2.6E-04	−4.2E-03
β_32_	–	−2.4E-06	4.3E-03
**IN-SAMPLE TEST**			
*R*^2^	0.548	0.571	0.551
Adjusted *R*^2^	0.535	0.544	0.524
*P*-value	<2.2E-16	6.3E-16	5.2E-15
**OUT-OF-SAMPLE TEST**			
*R*^2^	0.545	0.624	0.555

**Figure 5:**
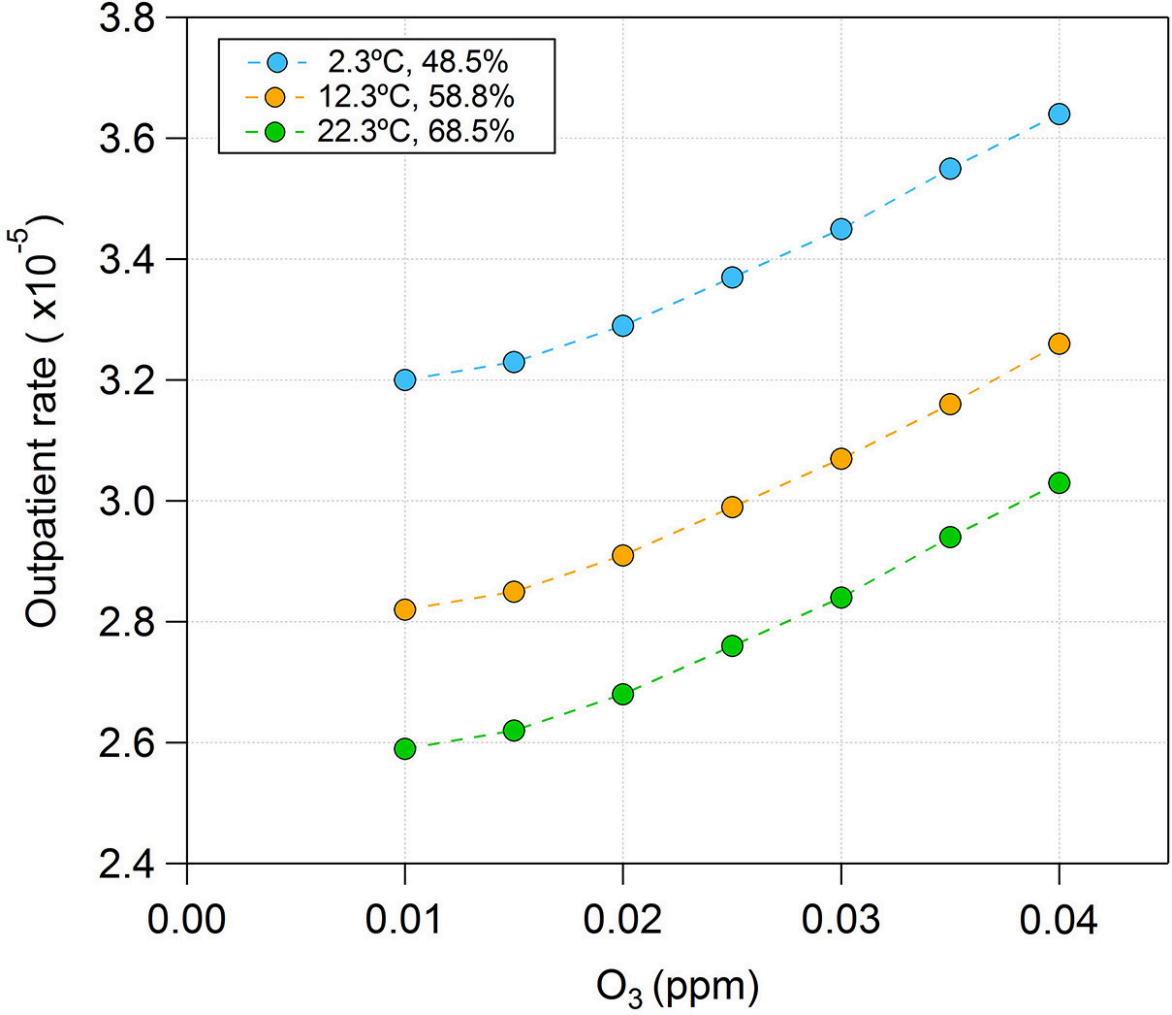
An example of predicted outpatient rate by model 2.

The model 2 can predict the outpatient rate with temperature, humidity, and O_3_. The out of sample test shows the prediction accuracy of the regression model since the sample for out of sample test does not use for model development. The Figure 5 shows an example of the outpatient rate prediction with the model 2. Figure 5 shows the estimated outpatient rate by model 2 for three different temperature and humidity combinations (Temperature, Humidity) over O_3_. In South Korea, temperature and humidity increase during the summer and decrease during the winter. The three temperature and humidity combinations, high, average, and low, were determined based on the average temperature and humidity over the test time periods; these were 12.34°C and 58.5%, respectively. The outpatient rate increased with increased temperature and humidity. In contrast, the dry eye disease outpatient rate increased with reduced relative humidity (Hwang et al., 2016). This is presumably due to multiple factors rather than the simple effect of relative humidity. The regression models including sex and age, were developed based on model 2. The additional independent variables for the regression model were defined as follows:

1. Sex: 0 for male and 1 for female,
2. Age: 1 (0–9 years old), 2 (10–19 years old), 3 (20–29 years old), …, 9 (> 80 years old).

**Figure 6:**
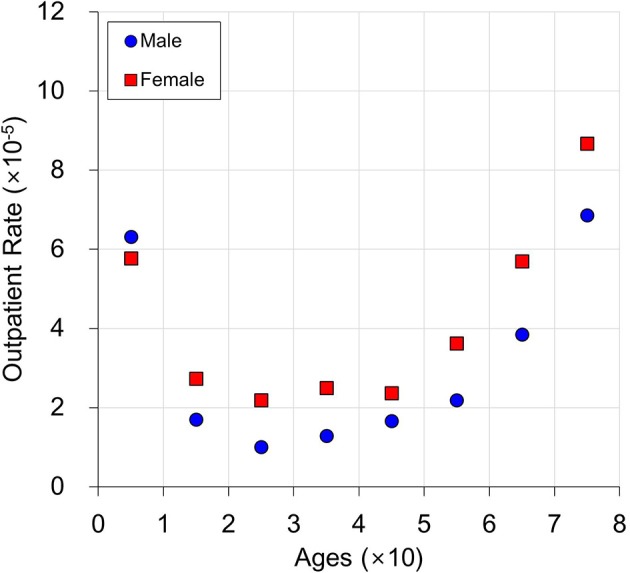
An example of predicted average outpatient rate.

Figure 6 shows the average outpatient rate over 156 weeks for each sex and age. The outpatient rates decrease until the 20–29 years old group, then typically increase for the younger ages for both males and females. The female outpatient rates are higher than those for males, for all age ranges except 0–9 years old.

Regression models were developed for each sex and age combination, as shown in Table 2. However, sex and age can be independent variables by assuming each coefficient of model 2 is a function of sex and age.

**Table 2:** Regression models for each sex and each age by using model 2.

**Sex**	**Age**	**β_0_**	**β_11_**	**β_12_**	**β_21_**	**β_22_**	**β_31_**	**β_32_**
0	1	−2.0*E*−05	2.9*E*−06	−5.0*E*−05	−9.3*E*−07	4.0*E*−05	1.3*E*−03	−8.4*E*−06
0	2	−8.1*E*−05	1.4*E*−06	−2.1*E*−05	−9.3*E*−07	4.6*E*−05	−2.1*E*−04	7.6*E*−08
0	3	−1.2*E*−05	5.9*E*−07	−1.2*E*−05	−2.6*E*−07	1.1*E*−05	2.6*E*−04	−2.2*E*−06
0	4	−1.1*E*−04	2.7*E*−07	−1.7*E*−06	−8.2*E*−07	4.4*E*−05	−1.8*E*−04	2.9*E*−06
0	5	1.1*E*−04	−3.6*E*−08	3.9*E*−06	4.9*E*−07	−3.4*E*−05	1.5*E*−04	−8.2*E*−07
0	6	7.3*E*−05	1.5*E*−07	−4.0*E*−06	4.8*E*−07	−1.7*E*−05	2.6*E*−04	1.5*E*−06
0	7	2.5*E*−04	8.6*E*−07	−1.2*E*−05	1.2*E*−06	−8.2*E*−05	8.2*E*−04	−1.2*E*−05
0	8	4.8*E*−04	1.4*E*−06	−2.7*E*−05	1.3*E*−06	−6.9*E*−05	−1.2*E*−03	3.3*E*−05
0	9	6.6*E*−04	3.6*E*−06	−8.9*E*−05	1.8*E*−06	−5.7*E*−05	−3.5*E*−03	5.1*E*−05
1	1	1.3*E*−04	9.1*E*−07	−1.6*E*−05	1.2*E*−06	−6.5*E*−05	2.3*E*−03	−2.5*E*−05
1	2	−1.7*E*−04	3.6*E*−07	4.1*E*−06	−8.0*E*−07	4.8*E*−05	4.3*E*−04	−3.6*E*−06
1	3	−1.5*E*−05	6.4*E*−07	−7.0*E*−06	−2.5*E*−07	1.1*E*−05	7.94*E*−05	−2.5*E*−06
1	4	−1.5*E*−05	1.0*E*−06	−1.9*E*−05	−5.5*E*−07	2.2*E*−05	1.4*E*−04	−3.3*E*−06
1	5	8.6*E*−05	8.6*E*−08	3.7*E*−06	4.8*E*−07	−3.4*E*−05	3.4*E*−04	−5.6*E*−06
1	6	9.9*E*−05	3.3*E*−07	2.0*E*−06	3.4*E*−07	−3.6*E*−05	4.7*E*−04	−9.4*E*−06
1	7	5.3*E*−04	1.5*E*−06	−2.9*E*−05	2.0*E*−06	−1.4*E*−04	2.5*E*−04	−1.2*E*−06
1	8	8.2*E*−04	1.9*E*−06	−2.3*E*−05	3.4*E*−06	−2.3*E*−04	−4.5*E*−04	−2.0*E*−06
1	9	8.4*E*−04	4.8*E*−06	−1.3*E*−04	2.0*E*−06	−9.6*E*−05	−1.6*E*−03	4.6*E*−05

Assuming β_0_ and β_*ij*_ in Table 2 are functions of sex and age, then let the function be *g*_0_(*sex, age*) and *g*_*ij*_(sex, age); the regression model can be represented as follows:

$$\begin{array}{l} \text{y}=g_{0}(\text{sex},\text{age})+g_{11}(\text{sex},\text{age})\cdot X_{1}+g_{12}(\text{sex},\text{age})\cdot \text{ln}(X_{1})\\ \;+g_{21}(\text{sex},\text{age})\cdot X_{2}+g_{22}(\text{sex},\text{age})\cdot \text{ln}(X_{2})+g_{31}(\text{sex},\text{age})\cdot X_{3}\\ \;+g_{32}(\text{sex},\text{age})\cdot \text{ln}(X_{3})+\text{ ε} \end{array}$$

This is a multi-level regression model; thus, model 2 is a first-level regression model and *g*_0_(sex, age) and *g*_*ij*_ (sex, age) are second-level regression models (Gelman and Hill, 2007). This model is applicable when there is a hierarchical structure among independent variables. In this study, sex and age were considered higher-level independent variables. Because the effect of age is nonlinear, as shown in Figure 6, the regression model for *g*_0_(sex, age) was developed by the following relationship: sex + age + sex · age + ln (age) + sex· ln (age) + exp(age) + sex · exp(age). In order to develop a simple model, the model selected for *g*_*ij*_ (sex, age) was one of the following relationships:

1. sex + age + sex · age,
2. sex + ln(age) + sex · ln(age),
3. sex + exp(age) + sex · exp(age).

The model that provided the highest *R*^2^ value in Table 2, when β_*ij*_ was the response variable and sex and age were the independent variables, was selected. Two regression models were separately developed by age, because the effect of age dramatically changed between 20 and 30 years old, as shown in Figure 6. The first regression model for ages 1 and 2 is as follows (this model does not have any ln(age) and exp(age) because age has only two levels):

$$\begin{array}{ll} y=g_{0}\left(\text{sex},\text{age}\right)+g_{11}\left(\text{sex, age}\right)\cdot X_{1}\\ + & g_{12}\left(\text{sex, age}\right)\cdot \text{ln}\left(X_{1}\right)+g_{21}\left(\text{sex, age}\right)\cdot X_{2}\\ + & g_{22}\left(\text{sex, age}\right)\cdot \text{ln}\left(X_{2}\right)+g_{31}\left(\text{sex, age}\right)\cdot X_{3}\\ + & g_{32}\left(\text{sex, age}\right)\cdot \text{ln}\left(X_{3}\right)\\ g_{0}\left(\text{sex, age}\right)=3.340e-05+4.042e-04\cdot \text{sex}\\ - & 5.738e-05\cdot \text{age}-2.466e-04\cdot \text{sex}\cdot \text{age}\\ g_{11}\left(\text{sex, age}\right)=4.321\text{e}-06-2.865\text{e}-06\cdot \text{sex}\\ - & 1.463\text{e}-06\cdot \text{age}+9.143\text{e}-07\cdot \text{sex}\cdot \text{age}\\ g_{12}\left(\text{sex, age}\right)=-7.901\text{e}-05+4.214\text{e}-05\cdot \text{sex}\\ + & 2.891\text{e}-05\cdot \text{age}-8.402e-06\cdot \text{sex}\cdot \text{age}\\ g_{21}\left(\text{sex, age}\right)=-9.365\text{e}-07+4.096\text{e}-06\cdot \text{sex}\\ + & 3.474\text{e}-09\cdot \text{age}-1.984\text{e}-06\cdot \text{sex}\cdot \text{age}\\ g_{22}\left(\text{sex, age}\right)=3.37\text{e}-05-2.128\text{e}-04\cdot \text{sex}\\ + & 5.929\text{e}-06\cdot \text{age}+1.078\text{e}-04\cdot \text{sex}\cdot \text{age}\\ g_{31}\left(\text{sex, age}\right)=2.826\text{e}-03+1.282\text{e}-03\cdot \text{sex}\\ - & 1.519e-03\cdot \text{age}-3.189\text{e}-04\cdot \text{sex}\cdot \text{age}\\ g_{32}\left(\text{sex, age}\right)=-1.693\text{e}-05-3.024\text{e}-05\cdot \text{sex}\\ + & 8.502\text{e}-06\cdot \text{age}+1.331\text{e}-05\cdot \text{sex}\cdot \text{age} \end{array}$$

The second regression model for ages 3, 4, 5, 6, and 7 is as follows (Age 8 and 9 data were removed for model development because their data patterns differ from the others, likely due to the effects of old age):

$$\begin{array}{ll} y=g_{0}\left(\text{sex, age}\right)+g_{11}\left(\text{sex, age}\right)\cdot X_{1}\\ + & g_{12}\left(\text{sex, age}\right)\cdot \text{ln}\left(X_{1}\right)+g_{21}\left(\text{sex, age}\right)\cdot X_{2}\\ + & g_{22}\left(\text{sex, age}\right)\cdot \text{ln}\left(X_{2}\right)+g_{31}\left(\text{sex, age}\right)\cdot X_{3}\\ + & g_{32}\left(\text{sex, age}\right)\cdot \text{ln}\left(X_{3}\right)\\ g_{0}\left(\text{sex, age}\right)=-1.118\text{e}-06-4.366\text{e}-06\cdot \text{sex}-1.43\text{e}\\ - & 05\cdot \text{age}+1.658\text{e}-05\cdot \text{ln}\left(age\right)+3.153\text{e}\\ - & 07\cdot \exp\left(\text{age}\right)+1.303\text{e}-05\cdot \text{sex}\cdot \text{age}-2.749\text{e}\\ - & 05\cdot \text{sex}\cdot \text{ln}\left(\text{age}\right)+1.67\text{e}-07\cdot \text{sex}\cdot \text{exp(age)}\\ g_{11}\left(\text{sex, age}\right)=1.867\text{e}-07+2.569\text{e}-07\cdot \text{sex}+5.21\text{e}\\ - & 10\cdot \text{exp(age)}+2.57\text{e}-10\cdot \text{sex}\cdot \text{exp(age)}\\ g_{12}\left(\text{sex, age}\right)=-2.652\text{e}-06-1.15\text{e}-06\cdot \text{sex}-6.831\text{e}\\ - & 09\cdot \text{exp(age)}-1.041\text{e}-08\cdot \text{sex}\cdot \text{exp(age)}\\ g_{21}\left(\text{sex, age}\right)=-2.73\text{e}-07-2.182\text{e}-08\cdot \text{sex}+1.419\text{e}\\ - & 09\cdot \exp\left(\text{age}\right)+6.259\text{e}-10\cdot \text{sex}\cdot \text{exp(age)}\\ g_{22}\left(\text{sex, age}\right)=1.572\text{e}-05-4.796\text{e}-06\cdot \text{sex}-9.083\text{e}\\ - & 08\cdot \exp\left(\text{age}\right)-4.214\text{e}-08\cdot \text{sex}\cdot \text{exp(age)}\\ g_{31}\left(\text{sex, age}\right)=-4.051\text{e}-08+3.256\text{e}-04\cdot \text{sex}+1.339\text{e}\\ - & 04\cdot \text{age}-6.687\text{e}-05\cdot \text{sex}\cdot \text{age}\\ g_{32}\left(\text{sex, age}\right)=8.94\text{e}-06-9.927\text{e}-06\cdot \text{sex}-2.22\text{e}\\ - & 06\cdot \text{age}+1.538\text{e}-06\cdot \text{sex}\cdot \text{age} \end{array}$$

Table 3 shows the test results for the two developed regression models. The p values for both regression models were less than 2.2e-16; both models were statistically significant. In the in-sample tests, when ages were 1 and 2, *R*^2^ and adjusted *R*^2^ were 0.774 and 0.758, respectively. When ages were 3 through 7, *R*^2^ and adjusted *R*^2^ were 0.736 and 0.728, respectively. In the out-of-sample tests, when age was 1 and 2, *R*^2^ was 0.7; when age was 3 through 7, *R*^2^ was 0.753. This result shows that the model is valid. It is also possible to develop multi-level regression models with model 1 or model 3 in Table 1; these provide lower *R*^2^ than those by model 2. The regression models can predict conjunctivitis outpatient rate and perform sensitivity analysis for each independent variable. To predict the conjunctivitis outpatient rate by sex and age, model 1 can be used. Model 3 can be used to predict the conjunctivitis outpatient rate by temperature, humidity, and O_3_. Model 2, the multi-regression model, can be applied when all independent variables are combined to predict conjunctivitis outpatient rate.

**Table 3:** Test results for multi-level regression models.

	**Regression Model**	
	**Age: 1 and 2**	**Age: 3, 4, 5, 6, and 7**
**IN-SAMPLE TEST**		
*R*^2^	0.774	0.736
Adjusted *R*^2^	0.758	0.728
*p*-value	<2.2E-16	<2.2E-16
**OUT-OF-SAMPLE TEST**		
*R*^2^	0.748	0.753

**Figure 7:**
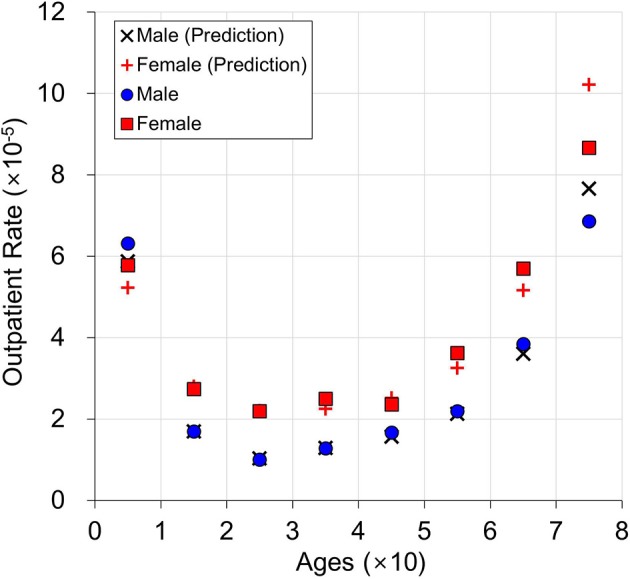
Prediction example for age and sex with average outpatient rate.

An example of multi-level regression model prediction is shown in Figure 7. The average temperature, average humidity, and average O_3_ (0.018 ppm) over 156 weeks were used for this graph. This is compared with the average outpatient rate in Figure 6. The average outpatient rates are close to predictions by the multi-level regression model. When age is 1, the male outpatient rate is higher than the female outpatient rate. In contrast, in all other age ranges, male outpatient rates are lower than female outpatient rates. The multi-regression model predicts the number of conjunctivitis outpatients based on age and sex, by using the weekly average temperature, humidity, and O_3_.

**Figure 8:**
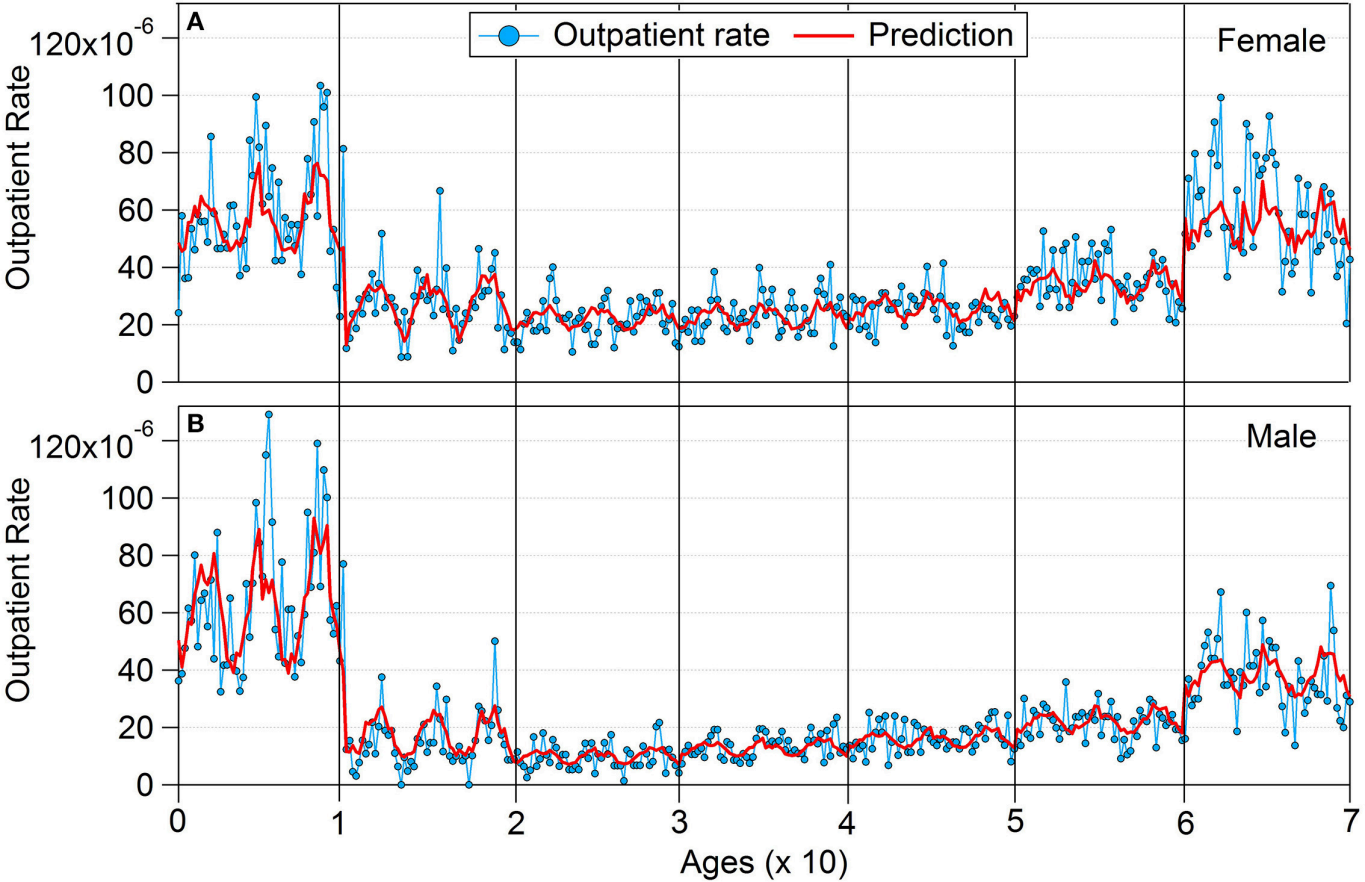
Prediction and actual outpatient rate using out-of-sample testing: **(A)** female and **(B)** male.

Figure 8 shows the comparison between prediction and actual outpatient rate by using out-of-sample tests. Fifty-two-week data for each sex and age were used for all 3 years. The overall prediction followed the individual trends, except for a large variation within age 7; this is presumably related to increased mortality in the age 7 group. These results indicate that the developed multi-regression model can predict the incidence of conjunctivitis by using age, sex, temperature, humidity, and O_3_. The level 1 regression model can predict the overall incidence of conjunctivitis without consideration of sex and age (Model 2 in Table 2).

May insert up to 5 heading levels into your manuscript as can be seen in “Styles” tab of this template. These formatting styles are meant as a guide, as long as the heading levels are clear, Frontiers style will be applied during typesetting.

## Conclusions

The weekly average O_3_ concentrations were highly correlated with meteorological factors and numbers of outpatients. This study provides models for prediction of conjunctivitis outpatient rates by using multiple concurrent independent variables, such as temperature, humidity, and O_3_. This model verifies the effect of O_3_ by the developed regression model. When O_3_ increases, the outpatient rate also increases. A method to develop a multi-level regression model for the conjunctivitis outpatient rate is provided. Sex and age factors are added to the developed regression model by using multi-level regression modeling. This enabled us to predict the conjunctivitis outpatient rate by using five independent factors concurrently. The developed models can be used to identify the characteristics of conjunctivitis outpatient rate on the basis of each independent variable. Test results for the developed models and their prediction examples are provided. Other pollutants can be included in future research. In future study, we will apply the multi-level regression model to other environmental diseases.

## Author contributions

SP and C-KJ supervised overall research. J-WS contributed to paper writing and model development. J-SY performed the air pollutant and meteorological data analysis.

### Conflict of interest statement

The authors declare that the research was conducted in the absence of any commercial or financial relationships that could be construed as a potential conflict of interest.
